# Childhood abuse is associated with methylation of multiple loci in adult DNA

**DOI:** 10.1186/1755-8794-7-13

**Published:** 2014-03-11

**Authors:** Matthew Suderman, Nada Borghol, Jane J Pappas, Snehal M Pinto Pereira, Marcus Pembrey, Clyde Hertzman, Chris Power, Moshe Szyf

**Affiliations:** 1 Sackler Program for Epigenetics & Developmental Psychobiology, McGill University, 3655 Promenade Sir William Osler, Montreal H3G 1Y6, QC, Canada; 2 Department of Pharmacology and Therapeutics, McGill University, 3655 Promenade Sir William Osler, Montreal H3G 1Y6, QC, Canada; 3 McGill Centre for Bioinformatics, McGill University, 3649 Promenade Sir William Osler, Montreal H3G 0B1, QC, Canada; 4 MRC Centre of Epidemiology for Child Health/ Centre for Paediatric Epidemiology and Biostatistics, UCL Institute of Child Health, 30 Guilford Street, London WC1N 1EH, UK; 5 Clinical and Molecular Genetics Unit, UCL Institute of Child Health, 30 Guilford Street, London WC1N 1EH, UK; 6 Human Early Learning Partnership, University of British Columbia, Suite 440, 2206 East Mall, Vancouver V6T 1Z3, BC, Canada

**Keywords:** Epigenetics, Childhood abuse, Early life environment, Epigenome, DNA methylation, Biomarker

## Abstract

**Background:**

Childhood abuse is associated with increased adult disease risk, suggesting that processes acting over the long-term, such as epigenetic regulation of gene activity, may be involved. DNA methylation is a critical mechanism in epigenetic regulation. We aimed to establish whether childhood abuse was associated with adult DNA methylation profiles.

**Methods:**

In 40 males from the 1958 British Birth Cohort we compared genome-wide promoter DNA methylation in blood taken at 45y for those with, versus those without, childhood abuse (n = 12 vs 28). We analysed the promoter methylation of over 20,000 genes and 489 microRNAs, using MeDIP (methylated DNA immunoprecipitation) in triplicate.

**Results:**

We found 997 differentially methylated gene promoters (311 hypermethylated and 686 hypomethylated) in association with childhood abuse and these promoters were enriched for genes involved in key cell signaling pathways related to transcriptional regulation and development. Using bisulfite-pyrosequencing, abuse-associated methylation (MeDIP) at the metalloproteinase gene, *PM20D1*, was validated and then replicated in an additional 27 males. Abuse-associated methylation was observed in 39 microRNAs; in 6 of these, the hypermethylated state was consistent with the hypomethylation of their downstream gene targets. Although distributed across the genome, the differentially methylated promoters associated with child abuse clustered in genome regions of at least one megabase. The observations for child abuse showed little overlap with methylation patterns associated with socioeconomic position.

**Conclusions:**

Our observed genome-wide methylation profiles in adult DNA associated with childhood abuse justify the further exploration of epigenetic regulation as a mediating mechanism for long-term health outcomes.

## Background

Abuse in childhood, encompassing physical, sexual or emotional abuse, is a key component of a broader spectrum of child maltreatment [1]. Life-long consequences of child abuse have been identified, including a greater risk of violence and delinquency, as well as adult depression and attempted suicide [1]. Hazardous behaviors, such as smoking and alcoholism, have also been found to be associated with abuse in childhood [2-4] along with later disease risk factors, including obesity [1,5], poorer immune function [1,6-8] earlier menarche [9-11] and outcomes such as ischemic heart disease [6,12,13] and chronic obstructive lung disease [13,14]. Explanations including biological mechanisms for long-term outcomes of child abuse have yet to be fully explored.

DNA methylation and histone modification play crucial roles in development, adaptation and response to environmental signals [15]. Methylation of cytosine bases occurs at CpG sites and, in gene promoters, usually results in gene silencing, whereas loss of methylation is associated with activity. MicroRNAs that repress the expression of their often numerous target genes are also part of epigenetic regulation [16]. MicroRNAs can down regulate key players in the epigenetic regulation machinery, but can also be silenced themselves by DNA methylation [17]. Whilst epigenetic regulation, by definition, does not alter DNA sequence, DNA variants can influence methylation levels. However, DNA methylation associated with early adversity (prenatal famine) was found to be independent of that associated with genetic variation [18]. Evidence to date suggests that stable changes in DNA methylation in the hippocampus of humans [19] and rats [20,21] are triggered by maltreatment in early life.

Much DNA methylation is tissue specific [22] but most tissues are unavailable for population studies of living individuals. Given the multiple outcomes for childhood abuse, we hypothesize that DNA methylation associated with childhood abuse is system-wide [23]. Several recent studies have supported the possibility of differential DNA methylation associations with social adversity in peripheral blood cells. For example, Borghol et al., demonstrated association of DNA methylation profiles with early life socioeconomic position in blood cells [24]. Provencal et al., showed that differential maternal rearing is associated with differential DNA methylation profiles in both prefrontal cortex and blood T cells [25]. Klengel et al., demonstrated childhood trauma-dependent DNA demethylation in functional glucocorticoid response elements of *FKBP5* in blood cells [26]. Mehta et al., have delineated recently DNA methylation signatures of child trauma and posttraumatic stress disorder in blood cells [27]. Although blood cells turn over, they are derived from stem cells and progenitors that stay with us for a life long. Thus, it is plausible that a DNA methylation event in a stem cell population that is introduced in early life remains into adulthood.

We therefore aimed to establish whether childhood abuse is associated with adult gene promoter methylation in a genome-wide investigation of peripheral blood cells [24]. We studied 40 adult males enrolled in the 1958 British Birth Cohort who have been found to have substantial variation in promoter methylation in over 6,000 genes, with a distinct methylation profile associated with socio-economic position [24]. Those with childhood abuse in this cohort have been shown to have long-term associations with negative health outcomes, specifically, a greater prevalence of obesity among those who reported physical abuse in childhood [28].

## Methods

### Ethics statement

All participants provided written consent and a blood sample for DNA analysis; ethical approval for a 45y biomedical survey and data analysis was given by the South-East Multi-Centre Research Ethics Committee(ref. 01/1/44) and the Joint UCL/UCLH Committees on the Ethnics of Human Research (Committee A) (ref. 08/H0714/40).

### Study population

The selection of 40 adult males from the 1958 cohort [29] has been described previously [24] and are detailed in the Additional files. In brief, 17,638 participants were enrolled, all born in England, Scotland and Wales, during a single week in March 1958. At 45y, 4,177 males provided written consent and a blood sample for DNA analysis; ethical approval was given by the South-East Multi-Centre Research Ethics Committee. After exclusions (e.g. cancer or elevated C-reactive protein levels, immigrants), 3,362 white males were classified by socioeconomic position (SEP) and childhood abuse. Forty males were selected from extremes of SEP, including 12 who reported abuse (7 low and 5 high child SEP; 7 low and 5 high adult SEP). With exclusion of immigrants, the 1958 cohort shows little genetic population stratification [30].

Abuse was identified through participants' reports in a confidential questionnaire at 45y on the following experiences to age 16y: [1] "I was verbally abused by a parent"; [2] "I suffered humiliation, ridicule, bullying or mental cruelty from a parent"; [3] "I was physically abused by a parent –punched, kicked or hit or beaten with an object, or needed medical treatment"; [4] "I was sexually abused by a parent". A report of any of these was scored as abuse. These questions were from the PATH Through Life Project including items derived from the Parental Bonding Instrument, the British National Survey of Health and Development and the US National Comorbidity Survey [31].

### Measurement of relative DNA methylation levels

DNA sample preparation, methylated DNA immunoprecipitation (MeDIP) and microarray hybridization, scanning and data extraction were performed as described previously [24]. Briefly, DNA was extracted from whole blood collected in EDTA at 45 years using an in-house, manual guanidine hydrochloride and ethanol precipitation method. DNA promoter methylation data from 20,533 genes and 489 microRNAs for the 40 participants were generated using MeDIP with an antibody that recognizes and binds 5-methylcytosine (DNA methylation) to isolate methylated DNA fragments. These fragments were then hybridized to custom-designed, high-density oligonucleotide microarrays, covering approximately 1000 bp upstream to 250 bp downstream at 100 bp spacing from the transcription start sites (TSS) in Ensembl (version 44). Microarray data files used in this study can be downloaded from the Gene Expression Omnibus (accession number: GSE31713). Three replicate microarrays were generated per individual and demonstrated adequate reproducibility [24]. Both hierarchical clustering and principal components analysis applied to the 500 most variable probes across all microarrays showed that the three replicates clustered. Furthermore, >70% of the variance in these probes was explained by individual variation.

### Microarray statistical analysis

**Figure 1:**
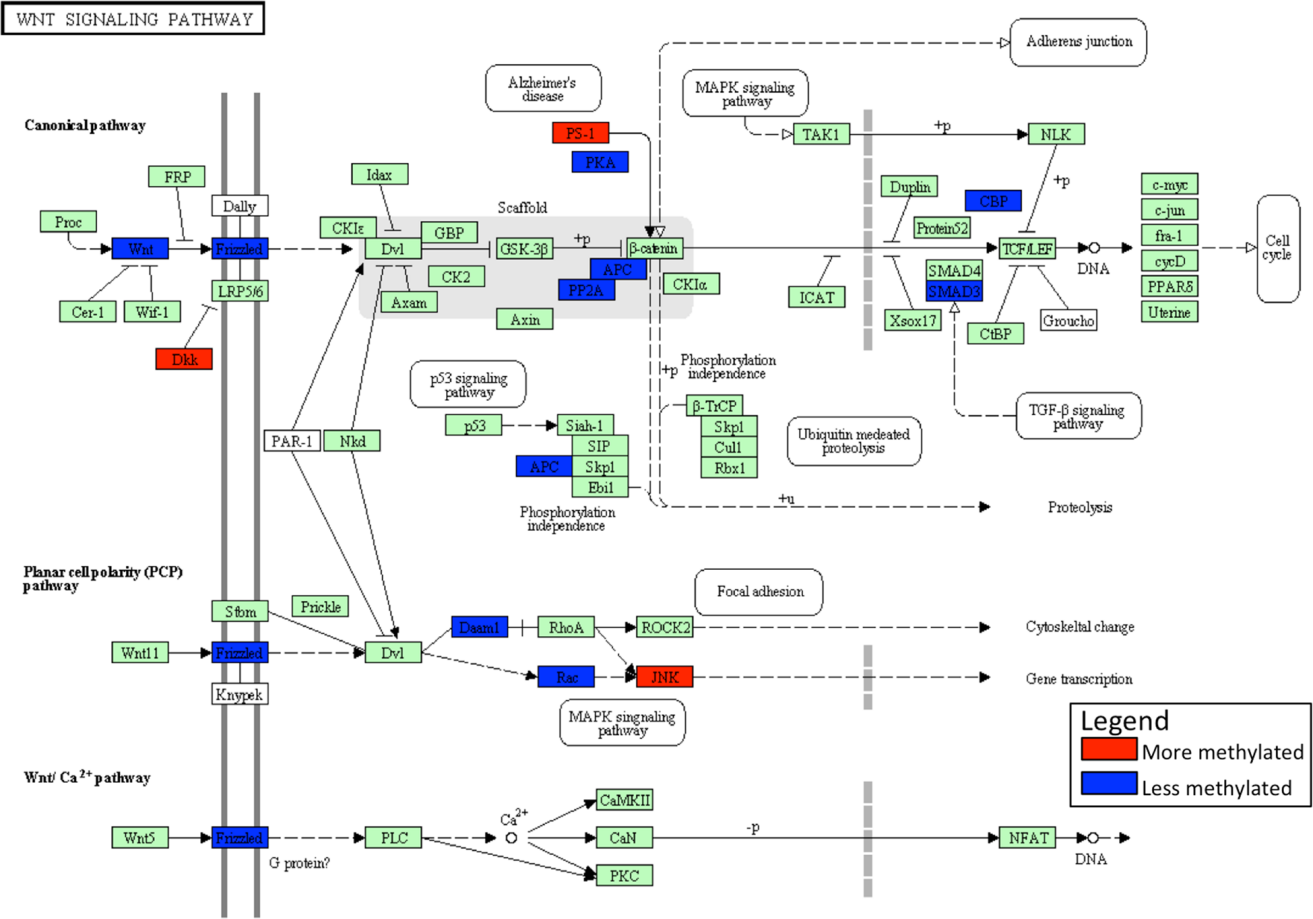
**Promoter methylation associated with childhood abuse.** Heatmap showing MeDIP probe values from the 997 differentially methylated promoters (rows) across all 40 participants (columns). Each promoter is represented by the probe most associated with childhood abuse. Blackened squares above the columns denote non-abuse males, white squares denote those with childhood abuse. Other covariates included are childhood and adulthood socio-economic position (white = low, gray = high). Neither appears to explain the main sample clusters.

The steps taken in the microarray statistical analysis are shown in Additional file 1: Figure S1 and justification for our approach is given in Additional files. Quality control involved generating MvA plots (i.e. plots of log(Cy5/Cy3) vs log(Cy5 × Cy3)) to identify those with severe dye biases or low signal. Microarrays deemed unacceptable were repeated, so no sample was excluded by quality control. Unsupervised clustering failed to identify batch effects related to hybridization date. Normalization of the final set of microarrays proceeded by computing log ratios of the bound (Cy5) and input (Cy3) microarray channel intensities for each microarray and then microarrays were normalized to one another using quantile normalization under the assumption that all samples have identical overall methylation levels. A probe was called differentially methylated if the modified t-statistic from ‘limma’ [32] of Bioconductor [33] was significant (p < 0.05) and the log2 fold-difference of the mean group probe intensities was ≥0.25. A promoter was called differentially methylated if it contained a probe called differentially methylated and if it contained probes for which modified t-statistics were significantly higher or lower than the average probe on the microarray. Significance for the latter was determined by applying the Wilcoxon rank-sum test and then calculating a corresponding false discovery rate (FDR) [34] using the method of Benjamini and Hochberg [35]. Promoters with FDR < 20% were called differentially methylated. This false discovery rate (FDR) was designed to test the chances of an overall false discovery among a series of related results. It is particularly useful for an exploratory analysis concerned with making general inferences from among a set of 'discoveries', rather than guarding against one or more individual false positives. The FDR threshold of 20% used here indicates that the expected proportion of promoters incorrectly called differentially methylated is around 20%. We find this threshold acceptable because this preliminary study is not meant to definitively characterise the epigenetic signatures of childhood abuse. In Figure 1, we present a heatmap showing probe methylation scores averaged across triplicate microarrays. Clustering was performed using Ward's hierarchical clustering algorithm with Pearson correlation distance as the distance metric.

All bioinformatic functional analysis was based on gene sets from GO [36], KEGG [37] and mSigDB [38]. Enrichment for differential methylation was determined by applying the hypergeometric test to the overlap between known gene sets and those found in our study to be differentially methylated. FDR values were obtained by adjusting these significance levels over all gene sets and pathways considered. The differentially methylated gene set was then subjected to pathway analysis using Ingenuity Pathway Analysis software (http://ingenuity.com/products/pathways_analysis.html).

In assessing megabase regions of the genome, methylation patterns were obtained by computing the mean methylation score difference between abuse and non-abuse groups for each probe, generating a UCSC wiggle track file from these differences and then uploading it for display on the UCSC genome browser (http://genome.ucsc.edu/).

### Validation and further methylation analysis

First, we validated the microarray calls, selecting 11 genes with the strongest methylation association with abuse (Additional file 2: Figure S2). Validation was performed using quantitative PCR (qPCR) of bound and input fractions of MeDIP with primers flanking the differentially methylated regions (Additional file 3: Table S1). Second, we validated two of these 11 genes, SLC17A3 and PM20D1, hypermethylated in association with abuse on MeDIP, by bisulfite pyrosequencing (in participants with sufficient DNA), as an independent method that measures methylation at specific sites [39]. Next, bisulfite pyrosequencing analysis of PM20D1 was repeated on an additional 27 males selected using the same criteria as the original [40] group. Details of pyrosequencing conditions, including optimization of PCR amplification using 0, 50 and 100% methylation controls are provided in Additional files.

Cell type ratios in blood are known to fluctuate so certain methylation differences between individuals could be caused by different cell ratios, particularly in promoters of genes with cell-type specific methylation. To rule out this possibility in our analysis, we compared our results to published MeDIP [40], expression [41] and Illumina 450 K [42] profiles of purified blood cell types. In each published dataset, we identified all differentially methylated or expressed genes or probes (as appropriate) between all pairs of available blood cell type profiles and then compared those lists of differences to the list of differentially methylated genes or probes between the abused and non-abused individuals in our study. If variation in blood cell type ratios explains the methylation differences in our analysis, then we would expect to see at least one larger-than-expected intersection. In each case, however, hypergeometric tests failed to identify larger-than-expected intersections (p > 0.4 in each case). For the published MeDIP dataset [40], the microarray design used was similar to our design so we were able to construct a 1-1 mapping between over half of the probes across our respective designs. Probes were paired if they were closest and within 150 bp. Unfortunately, this MeDIP dataset only contained profiles for B and T cell purified cell types. We therefore expanded our analysis to include an expression dataset [41] with profiles for CD33+ (myeloid), CD34+, CD71+ (early erythroid), CD4+, CD8+, CD14+ (monocyte), CD19+ (B) and CD56 (natural killer) cells. We also included a recent Illumina 450 K dataset [42] with profiles for granulocytes, neutrophils, eosinophils, CD4+, CD8+, CD14+, CD19+ and CD56+ cells. For both these datasets, results were compared at the gene level.

## Results and discussion

Physical, cognitive and emotional characteristics and biomarkers are listed for participants in Table 1. As expected, the abuse group showed more adverse characteristics than the non-abuse group, but differences did not reach conventional p-values in this small sample.

**Table 1:** Characteristics of the 40 male study participants

	**Age (y)**	**No abuse n = 28**	**Abuse n = 12**	p*
Birthweight, g, mean ± SD^#^	0	3577.35 (574.91)	3338.21 (590.25)	0.24
Height, cm, mean ± SD^#$^	7	1.24 (0.07)	1.21 (0.07)	0.27
Maths score, mean ± SD^#$^	16	14.82 (7.32)	12.29 (7.95)	0.44
Reading score, median (Q1, Q3)^#$^	16	27 (21, 31)	31 (12, 32)	0.70
Socio-emotional adjustment number ~ median (Q1-Q3)^#$^	7	4 (1, 12)	8.5 (2, 13)	0.47
Alcohol drinks daily, n (%)^#^	42	7 (25.93)	2 (16.67)	0.53
Smokers, n (%)^#^	42	7 (25.93)	4 (33.33)	0.64
Height, cm, mean ± SD^#^	42	1.78 (0.09)	1.76 (0.06)	0.52
BMI, kg/m^2^, mean ± SD	45	26.63 (3.99)	28.69 (4.39)	0.16
Waist circumference, cm, mean ± SD	45	97.43 (10.24)	102 (12.02)	0.23
Diastolic blood pressure, mmHg, mean ± SD	45	82.77 (11.71)	85.53 (12.72)	0.51
Systolic blood pressure, mmHg, mean ± SD	45	132.92 (18.61)	134.72 (18.90)	0.78
Fev1^†^, mean ± SD^#^	45	3.84 (0.65)	3.70 (0.63)	0.53

^†^FEV1 = one-second forced expiratory volume; best test of three spirometry readings.

^#^N for non-abuse <28 (range 22 to 27).

^$^N for abuse <12 (range 7 to 10).

~higher score = poorer adjustment.

*p-value from t-test, except for median (IQR), when Two-sample Wilcoxon rank-sum test was used.

### Hundreds of promoters are differentially methylated in association with child abuse

In total, 997 gene promoters were differentially methylated in association with childhood abuse, affecting 1141 different genes (Additional files). Of these promoters, 311 were hypermethylated and 686 were hypomethylated in abused compared to non-abused males. Figure 1 shows a heatmap depicting the relative methylation levels for all differentially methylated promoters and how they cluster within study participants. Even at more stringent thresholds (p < 0.01 and q < 0.05, see Methods), there were still 34 differentially methylated promoters corresponding to 58 different genes with similar proportions hypermethylated to hypomethylated. These cluster the study participants very similarly to the larger set of differentially methylated promoters (Additional file 4: Figure S3). To assess whether the broad methylation signature of childhood abuse was affected by the numerical imbalance of abused versus non-abused (N = 12 vs 28), we conducted a permutation analysis. We found that 997 differentially methylated promoters between abused and non-abused was larger than the number of differences associated with 82% of random partitions (410 of 500) of the participants with partition size ratios corresponding to 12 vs 28. To address any concern that the abuse associated methylation differences were reflecting differences in blood cell type ratios, we compared our results with recently published expression and methylation profiles of purified cell types [40-42]. We found no evidence of statistically significant overlaps between our results for abuse and cell-type specific methylation and expression patterns (p > 0.4, hypergeometric test; see Methods for details).

**Figure 2:**
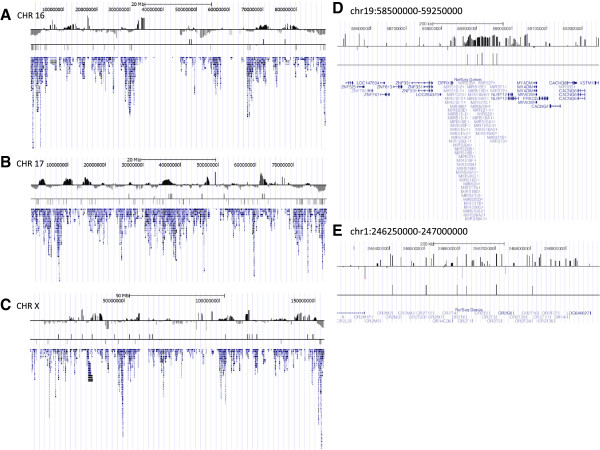
**Validation of MeDIP results. A**. Quantification of methylation differences in the abuse and non-abuse groups by bisulfite pyrosequencing analysis of the *SLC17A3* promoter and the *PM20D1* first exon and intron. DNA methylation at 14 CpG sites in the *SLC17A3* promoter and 12 and 1 CpG sites in the *PM20D1* first exon and first intron, respectively, among the abuse and non-abuse groups is shown (N = 10 vs. 26 for *SLC17A3*; N = 9 vs 23 for *PM20D1*). One-sided t-tests were applied to each CpG site to test for association of methylation levels with childhood abuse, and false discovery rates were calculated for the resulting p-values in order to correct for multiple testing. All false discovery rates (FDR) were less than 0.1, indicating significant association between CpG methylation levels and childhood abuse. **: FDR < 0.025; *: FDR < 0.05; ++: FDR < 0.1; +: FDR < 0.2. The bars represent average methylation for all subjects in a group and error bars indicate the standard error of the mean. Physical maps of the regions analyzed are presented above the charts where CpG positions are indicated by balloons. The transcription start site (TSS) is indicated by a hook arrow. The positions of the primers used for pyrosequencing (Additional file 3: Table S2) are indicated by arrows. **B**. Replication of the quantification of the differences in methylation at *PM20D1* between the abuse and non-abuse groups in an additional 27 males that were not profiled using MeDIP (N = 7 vs. 20). Pyrosequencing was applied to measure the methylation levels of 13 CpG sites in the first exon and intron of *PM20D1*.

In 11 genes selected for validation, the direction of abuse associated methylation differences was confirmed using qPCR of bound and input MeDIP fractions (Additional file 2: Figure S2). We also confirmed abuse associated hypermethylation by pyrosequencing of sites in the promoter of *SLC17A3* and the first exon of *PM20D1* in the original samples (Figure 2A) and in an additional 27 males for *PM20D1* (Figure 2B), and with *SNP rs11540014* showing no association with methylation levels (data not shown). However, the associations in the promoter of *SLC17A3* were not replicated in the additional 27 males.

### Abuse-associated methylation clusters by biological function

**Figure 3:**
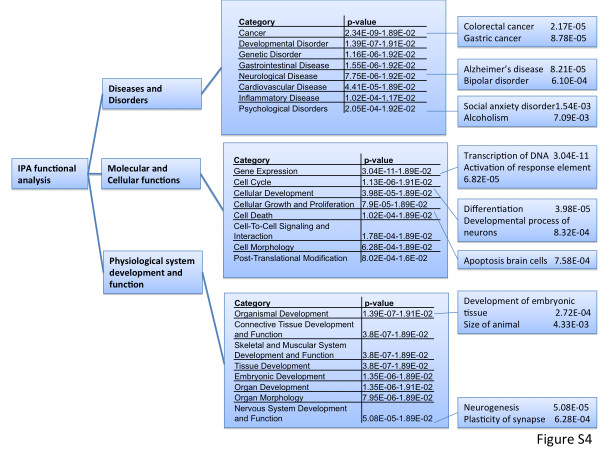
**Differential methylation in the *WNT *signaling pathway.** The KEGG (http://www.genome.jp/kegg/mapper.html) depiction of the *WNT* signaling pathway is shown with hypermethylated gene promoters (more methylated in the group with childhood abuse) colored red and the hypomethylated gene promoters colored blue.

Full results of functional analysis are given in Additional files. Differentially methylated gene promoters in abused males (1141 genes) were enriched in regulatory (169 genes) and developmental (230 genes) functions (Table 2). Central to both of these functions is the KEGG *WNT* signaling pathway; enriched for genes [15] for which promoters are hypomethylated in abused individuals, consistent with activation of this pathway in blood cells of the abuse group (Figure 3). No other KEGG pathway was enriched with differentially methylated genes at p <0.05 (uncorrected for multiple testing). Of the differentially methylated genes that perform some regulatory function, most (134 of 169) are hypomethylated in abused males. The regulation mainly affects transcription as indicated by enrichment of these genes in functional categories such as chromatin modification (28 genes), histone modification (11 genes) and transcription factor binding (35 genes). Similarly, most of the 230 developmental genes are hypomethylated in abused males (172 genes), best characterized by the general gene ontology category “multicellular organismal development” (163 genes). More specific subcategories do not show significant enrichment.

**Table 2:** Selected functional analysis of abuse associated hypo- and hypermethylation

**Pathway/function**	**Number of genes in pathway/function**	**Differentially methylated**		**Hypo-methylated**			**Hyper-methylated**		
		**n**	**p**	**n**	**p**	**fdr**	**n**	**p**	**fdr**
WNT signaling pathway	142	19	0.0013	15	0.0020	0.53	4	0.22	1
Regulation	2330	169	0.017	134	0.0018	0.51	33	0.88	1
- Chromatin modification	273	32	0.0004	28	0.00005	0.09	4	0.68	1
- Histone modification	105	13	0.013	11	0.008	0.94	2	0.53	1
- Transcription factor binding	493	41	0.034	35	0.006	0.84	6	0.84	1
Development	3054	230	0.0007	172	0.00096	0.40	58	0.17	1
- Multicellular organismal development	2838	213	0.0012	163	0.0006	0.32	50	0.32	1
Cell surface receptor linked signal transduction	1778	125	0.071	79	0.60	1	46	0.002	0.53

‘n’ is the number of genes in the relevant pathway that are differentially methylated in association with abuse.

‘p’ was calculated using the hypergeometric test, it indicates the statistical significance of the enrichment.

‘fdr’ the false discovery rate (FDR) corresponding to the p-value.

Differentially hypermethylated gene promoters in abused males are enriched in few functional categories. One of these, "cell surface receptor linked signal transduction", contains 125 genes with differentially methylated promoters of which 46 are hypermethylated in abused individuals. An Ingenuity functional analysis of the differentially methylated genes revealed similar molecular and cellular functions associated with transcriptional control (Additional file 5: Figure S4).

### Abuse-associated methylation consistent with microRNA targeting

MicroRNA genes are, like DNA methylation, known to repress the expression of target genes. However, unlike an individual methylation mark which typically targets a single nearby gene, each microRNA is associated with a specific set of a few hundred target genes [43]. We discovered an association of microRNA DNA hypermethylation with abuse. Of 489 microRNAs analysed, 39 were differentially methylated, of which 31 were hypermethylated in association with abuse. The target genes of six of these included a highly non-random proportion of genes with decreased promoter methylation in abused males (Table 3).

**Table 3:** Methylation of microRNAs and their target genes

**MicroRNA**	**Number of targets**	**Number hypo-methylated**	**Number hyper-methylated**	**Hypomethylated targets**	**Enrichment p-value**	**MicroRNA methylation**
mir-514	49	10	1	AFF4, BAALC, BRWD1, CARM1, ENAH, KLF13, MYO1B, NR3C1, SVIL, TCF12	5.71E-05	*hypermethylated*
let-7d	320	26	6	ATP2A2, BACH1, BRWD1, CDV3, CHD4, CPSF4, DCUN1D2, DOCK3, DOT1L, EFHD2, EZH2, GGA3, LIMD2, LRIG1, MECP2, MGAT4A, MIB1, MLL5, PARD6B, PBX3, PRTG, PTPRU, RDH10, SOCS1, UNC5A, WDR37	0.0030	*hypermethylated*
mir-520c	274	23	3	ASF1B, BCL2L11, BRP44L, DDHD1, DPYSL5, FLT1, FNDC3B, INHBB, KCNMA1, KLF13, MAP3K14, MECP2, MKNK2, MTUS1, ORMDL3, PBX3, PFN2, RGL1, SMAD2, UBE2Q2, WDR37, ZFP36L2, ZFPM2	0.0035	*hypermethylated*
mir-215	37	6	0	ARFGEF1, FNDC3B, GRHL1, KLHDC5, LRRFIP1, MECP2	0.0060	*hypermethylated*
mir-519a	377	28	4	AFF4, BRWD1, BTG3, CELSR2, DNAJB6, LRIG1, MAP3K5, MAP4, MASTL, MCM7, MECP2, MIB1, NPAS2, OBFC2A, PARD6B, PFN2, PTHLH, RAPGEF4, RASD1, SCAMP2, SFRS2, SMOC2, TMEM64, VGLL3, WHSC1, YES1, ZFPM2, ZFYVE9	0.0074	*hypermethylated*
mir-519e	104	11	2	ARHGEF12, ARL4C, BCOR, CCNG2, CTDSPL2, DLL1, DPYSL5, EFNB3, NEDD4L, NPAS2, RAB35	0.0075	*hypermethylated*
mir-203	239	20	3	AFF4, BCL7A, CNTFR, CTDSPL2, DNMT3B, EGR1, FALZ, INSIG1, KCTD9, LASP1, MECP2, PLD2, PPM1B, RAPGEF4, SLC12A2, SMAD1, SPEN, SPIRE1, TCF12, YWHAQ	0.0064	*hypomethylated*

MicroRNAs are listed that have statistically significant MeDIP differences between abuse and non-abuse groups whose predicted gene targets are enriched for gene promoters that are also differentially methylated between abuse and non-abuse groups. In each case, enrichment is for targets with lower methylation in the abused group.

“enrichment p-value” indicates the level of enrichment for hypomethylated targets.

“microRNA methylation” indicates whether the data predicts significantly higher (“hypermethylated”) or lower (“hypomethylated”) methylation levels in the abuse group.

### Abuse-associated hypomethylation and CpG density

DNA methylation in regions of relatively high CpG frequency, known as CpG islands, plays an important regulatory role in the otherwise CpG-depleted (≤40% of that expected) mammalian genome [44,45]. In spite of the fact that MeDIP is known to enrich for methylation differences away from CpG islands [46], we observed unusually high CpG frequencies in promoters with reduced methylation levels in abused individuals. This frequency (0.86) is significantly higher than that observed in the average promoter (frequency = 0.42; p < 1.4 × 10^-285^) as well as promoters with increased methylation levels in abused individuals (frequency = 0.38; p < 4 × 10^-138^; Additional file 6: Figure S5). This frequency (0.86) is even higher than the 0.6 threshold used to define CpG islands.

### Abuse-associated methylation clusters by genomic location

**Figure 4:**
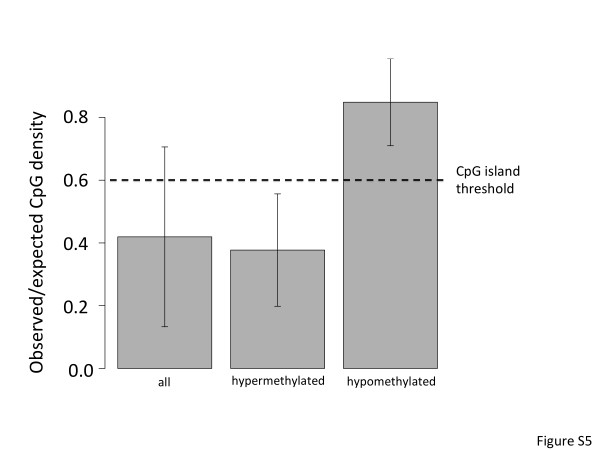
**Megabase regions enriched with methylation differences.** Promoter MeDIP differences across chromosomes 16 **(A)**, 17 **(B)** and X **(C)** and across two smaller genomic regions **(D)** and **(E)** are shown using images obtained from the UCSC Genome Browser. The top track depicts average differences of log abuse – log non-abuse. Each bar in the middle track identifies a significant difference. Bars above or below the horizontal line identify sites with higher or lower methylation in the abused group. The bottom track indicates relative gene abundance across the chromosome.

Differentially methylated DNA loci associated with early life environments tend to cluster in the genome [24,47]. Chromosome-wide views of our data reveal megabase-sized regions significantly enriched for differentially methylated promoters (Figure 4). At the chromosomal level, chromosomes 16 and 17 were significantly enriched for hypomethylated promoters in abused individuals, whereas chromosome X was significantly enriched for hypermethylated promoters. At the megabase level, three regions were significantly enriched for differentially methylated promoters (p < 0.05). All were hypermethylated in abused individuals: chr1:246250000-247000000, chr14:100250000-101000000 and chr19:58500000-59250000 (genome assembly hg18), but only the regions on chromosomes 1 and 19 passed multiple testing correction with FDR below 0.2 (FDR < 0.006 and 0.0001, respectively; Figure 4D,E). The regions on chromosomes 14 and 19 each contain a cluster of microRNAs in which promoters account for all of their statistically significant site-specific differential methylation.

Clustering of differential promoter methylation, up to 2 Mb apart, was detectable across the entire genome (Additional file 7: Figure S6).

### Socio-economic position (SEP) and abuse

Previously, we identified 1252 gene promoters associated with childhood SEP and 545 associated with adulthood SEP [24]. Only 73 of 1252 (5.8%) and 19 of 545 (3.5%) gene promoters were also differentially methylated in association with childhood abuse. Just three (*CTAGE5*, *GNG4*, *MYO1B*) were differentially methylated in association with all three characteristics (childhood and adult SEP and childhood abuse). The association for *PM20D1* was specific to abuse.

## Conclusions

Blood DNA of 45y old males revealed differentially methylated gene promoters associated with abuse that occurred three decades earlier in childhood. There were several novel findings from our study. First, hundreds of specific promoter associations were uncovered, with approximately two-thirds hypomethylated in the abused group. Second, replication confirmed that hypermethylation in *PM20D1* is associated with childhood abuse. Third, microRNA gene targets tended to be hypomethylated, particularly when the microRNA itself was hypermethylated. Fourth, differentially methylated genes were clustered in discrete functional pathways and in genomic locations. These findings support the hypothesis that the differences in DNA methylation we observed were non-random and reflect an organized biological process.

It is now known that genes act through functional and interacting pathways, so we adopted a genome-wide approach to DNA methylation analysis, recognizing that modest epigenetic changes in numerous genes could reset the function of gene networks having phenotypic effects. We found enrichment of differentially methylated promoters in the *WNT* signaling pathway complex with hypomethylation of 15 genes in the abused. Elsewhere this pathway complex has been found to play a key role in embryonic development and cellular proliferation [48], and is deregulated in some chronic health conditions such as obesity [49-51], diabetes [52-54], metabolic syndrome [55], cancer [56-59] and inflammatory processes [56-58]. Whilst recognizing that our findings do not provide evidence for causal links between child abuse and later outcomes, they nonetheless raise the prospect of mediation by epigenetic modifications.

Of particular note was hypermethylation of *PM20D1* in association with abuse, given a previous study showing a variably methylated region at this metalloproteinase gene was hypermethylated in association with obesity [60]. This association persisted over 10 years of follow-up in an elderly population. Interestingly, child abuse has been shown to be associated with adult obesity in the full 1958 cohort [5] and is suggested by our Table 1. It is perhaps surprising to note that both our association with childhood abuse and the association with obesity were observed in blood DNA when *PM20D1* has its highest expression levels in the brain and lowest expression levels in blood [41]. Given that it is highly conserved from yeast to human, it likely plays a key though little understood role in the cell. By contrast, *SLC17A3* is like most of the genes differentially methylated in childhood abuse, most highly expressed in blood and a few specific brain regions (hypothalamus, prefrontal cortex, pituitary) [41]. It appears conserved in fewer species, mainly the higher mammals, and the expressed protein acts as a voltage-driven transporter in blood. Given this basic role, it is likely essential at nearly all stages of life.

Further support for epigenetic regulation working through interacting pathways comes from the striking enrichment in the abuse group of hypermethylated microRNAs combined with hypomethylation of their respective gene targets across the genome. It implies that during typical development, active transcription of these microRNAs is combined with synergistic target methylation to create a double layer of repression of these target genes; a repression that is lifted in association with child abuse.

Intriguingly, hypomethylated gene promoters in abused individuals typically contained sequences with very high CpG frequency. Demethylation of such CpG-rich promoters in abused males suggests that abuse leads to increased activity of key basic cellular functions, such as gene regulation and development, as found in pathway analysis. Another genomic feature associated with abuse was the clustering of differential promoter methylation detectable across the entire genome, providing further evidence of genome-wide as well as gene-specific organization of epigenetic profiles.

Previously we observed genome-wide clustering in association with SEP, but importantly, the “methylation signature” for abuse differed, such that <10% of the differentially methylated regions overlapped with childhood SEP [24]. Also, the differentially methylated genes were enriched in different functional pathways, notably, *MAP kinase* for SEP and *WNT* for child abuse. Further, the abuse associated differential methylation of microRNAs and their target genes was not seen for SEP. Whilst not ruling out generic associations with early life adversities, our findings suggest that different adversities are associated with different epigenetic changes to the genome.

Several methodological considerations arise here. First, reliable measurement of the frequency and severity of child abuse is not straightforward^1^. Child abuse was identified through participant’s report at 45y and was primarily emotional and physical abuse – only rarely sexual abuse. All measures have biases and inconsistencies yet retrospective reports are an accepted method of ascertainment in population studies [1]. Furthermore, prospective identification of abuse is not feasible in large studies and likely to be unrepresentative. By contrast, retrospective self-report, used here, is feasible though it is likely to underestimate true levels of abuse. Second, given the scale of assessing methylation at all promoters, we could only study a small but selected sample. Whilst our study is imbalanced with respect to abuse (12 vs 28) it has the benefit of control for SEP. Third, we used DNA from whole blood to test our hypothesis, currently the only practical option for population based studies. We cannot know the extent to which our results relate to gene expression. Use of whole blood also raises the possibility that abuse-associated differences in B-to-T cell ratios might account for some of our observations. We have partly addressed this by noting that B-cell and T-cell expression and methylation profiles [40,41] do not differ for many genes with abuse-associated methylation levels. Fourth, those abused in childhood might represent a distinct genetic group, but genetic differences alone are unlikely to account for all methylation differences observed here. Given the possibility of differences in epigenetic response due to genetic variation, future integrated studies of the epigenome and whole genome sequencing are an important next step. Fifth, our study is imbalanced including 28 controls compared to only 12 with childhood abuse resulting in reduced power to identify methylation differences. Nonetheless, this preliminary study was able to discover hundreds of differentially methylated promoters so future studies with better balance are likely discover many more. Finally, there is currently no ‘gold standard’ for measuring the methylome, yet MeDIP is a well-established genome-wide method that has been evaluated [46,61-65] and we confirmed all the micro-array calls in the top 11 methylation differences. Current genome-wide methods are more complementary than interchangeable and each has its strengths and weaknesses. Our analyses included triplicate arrays and methylation differences were confirmed in selected genes using other gene-specific methods both here and previously [24]. In using an analytic approach that was sensitive to subtle methylation associations across gene networks necessarily results in some false positives (for justification see Additional files). However, the non-random organization of methylation differences throughout the genome supports our main hypothesis that childhood abuse is associated with DNA methylation changes in adult blood.

In sum, the pattern of changes associated with child abuse detected in peripheral blood cells of 45 year-olds suggest that there is a system-wide readjustment of the epigenome to signals triggered by early life abuse. Our study does not demonstrate causality, nor can it demonstrate a temporal relationship between child abuse and DNA methylation levels in adulthood. It does, however, provide a justification for a range of studies addressing epigenetic responses to child abuse and their mediating role with later phenotypic outcomes.

## Competing interests

The authors declare that they have no competing interests.

## Authors’ contributions

Study was designed by CP, CH and MS. Participants were selected by CP and CH. Methylation analysis was completed by NB and JP. Bioinformatic analysis was performed by MS. The entire process was overseen by MS. JP, NB, CH, MP, SPP, CP, MS and MS all contributed to writing the manuscript. All authors read and approved the final manuscript.

## Pre-publication history

The pre-publication history for this paper can be accessed here:

http://www.biomedcentral.com/1755-8794/7/13/prepub

## Supplementary Material

Additional file 1: Figure S1 Summary of methods. Click here for file

Additional file 2: Figure S2 Validation by qPCR. Eleven gene promoters identified by microarray as being differentially methylated were subjected to real-time PCR quantification of the enrichment by the MeDIP procedure. Results were normalized against a methylated luciferase gene-containing plasmid (control), which was added to every sample in equal quantity before MeDIP. The y-axis represents relative concentration levels generated by applying PCR to methylation-enriched DNA. Each real-time PCR reaction was performed in duplicate for all subjects. Shown are the averages per group. Error bars indicate the standard error of the mean. Above the chart are tracks of the regions with the microarray data. (The bars indicate the difference between the abuse and the non-abuse groups, bars descending from the physical map are regions that are more methylated in the abused than the non-abused group; lower tracks identify probes with the most statistically significant differences). Primers for each PCR are given in Additional file 3: Table S1. They were selected so that the forward primer (denote by ‘F’) binds to the left and the reverse primer (denoted by ‘R’) binds to the right of the most significantly different probe. In some cases, two sets of PCR primers were designed, denoted by ‘set1’ and ‘set2’. 85% of the eleven gene promoters show statistically significant PCR quantification differences (*: P<0.05; **: P<0.01), hence validating differences found by microarray. Click here for file

Additional file 3 Supplementary Material. Click here for file

Additional file 4: Figure S3 Promoter methylation associated with childhood abuse. Heatmap showing MeDIP probe values from the 34 differentially methylated promoters (rows) across all 40 participants (columns) based on more stringent thresholds (q < 0.05 and p < 0.01, see Methods). Each promoter is represented by the probe most associated with childhood abuse. Blackened squares above the columns denote non-abuse males, white squares denote those with childhood abuse. Other covariates included are childhood and adulthood socio-economic position (white = low, gray = high). None appears to explain the main sample clusters. Click here for file

Additional file 5: Figure S4 Summary of functional analysis. Genes with hypermethylated or hypomethylated promoters in the abuse group were analysed by Ingenuity Pathway Analysis^**®**^. Gene categories enriched with this set of genes as well as enrichment p-values are listed. Click here for file

Additional file 6: Figure S5 CpG frequency in differentially methylated regions. Bars indicate average normalized CpG frequencies (observed/expected CpG frequency) of ‘all’ genomic regions profiled, regions ‘hypermethylated’ in abused individuals and regions ‘hypomethylated’ in abused individuals. Error bars depict standard deviation. The dashed line indicates the usual CpG frequency used to identify CpG islands. Click here for file

Additional file 7: Figure S6 Methylation dependencies across megabases. Shown are correlations of methylation differences from 500 kilobase regions at various distances apart. The level of clustering was quantified as the level of correlation between the differential methylation statistics within promoters at different distances apart. The solid grey region contains the 95% CI, and error bars contain the 95% CI for correlation values. Click here for file
