# Supplementary figures and images for: Childhood abuse is associated with methylation of multiple loci in adult DNA
Source: BMC Med Genomics. 2014 Mar 11;7:13. doi: 10.1186/1755-8794-7-13 (PMC4007631; doi:10.1186/1755-8794-7-13)

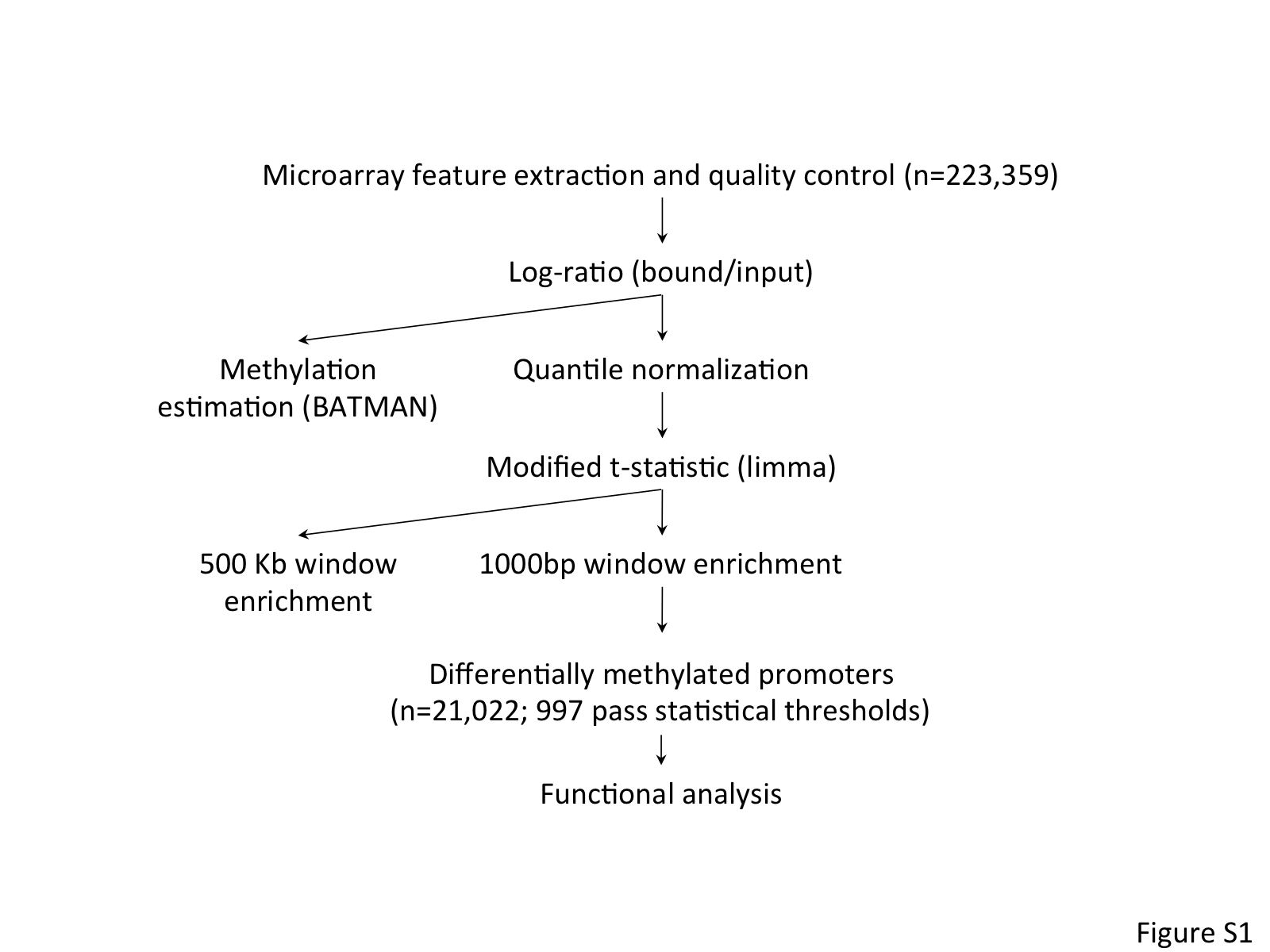

Supplement: Additional file 1: Figure S1 — Summary of methods. [file 1755-8794-7-13-S1.tiff]

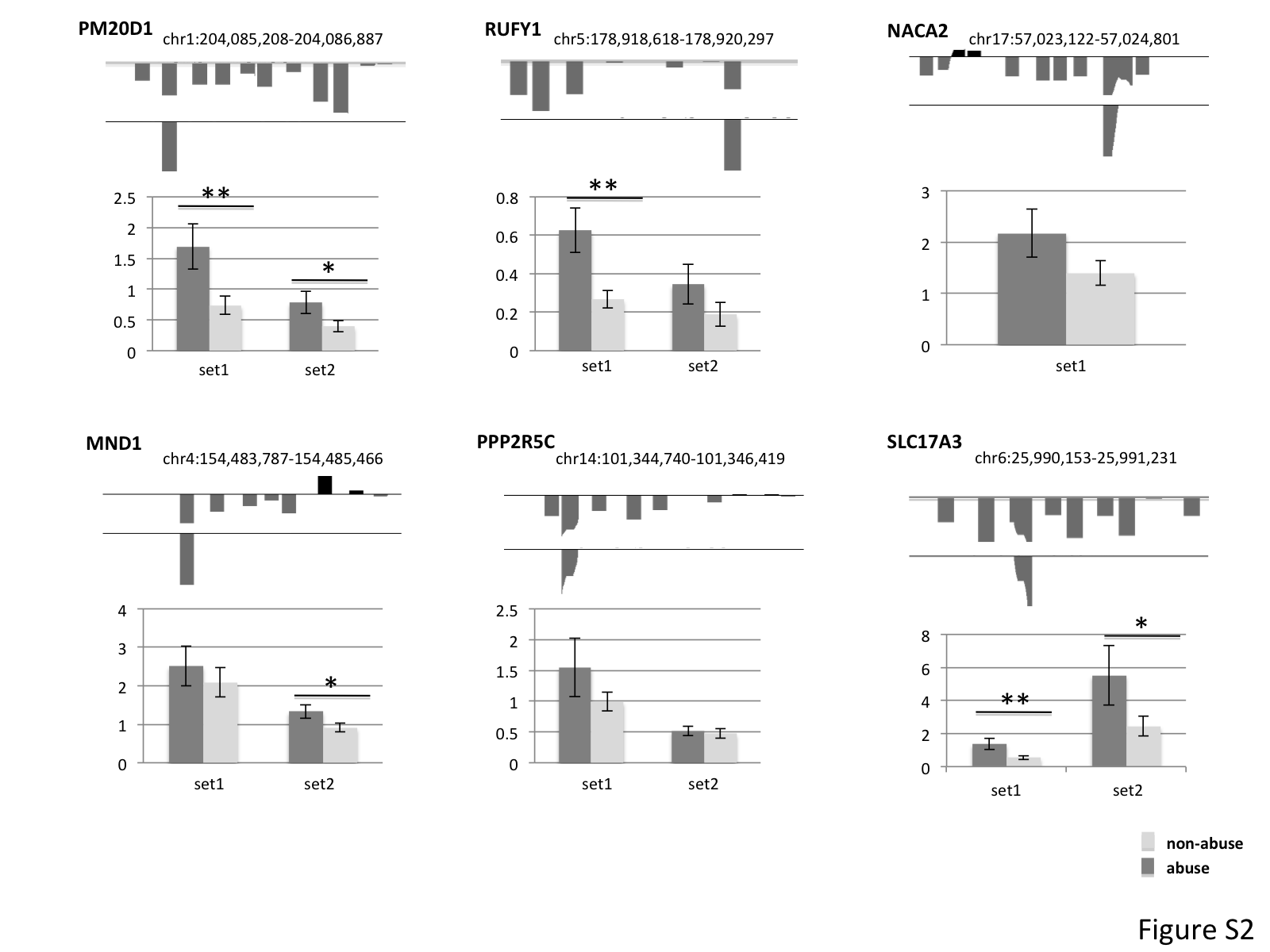

Supplement: Additional file 2: Figure S2 — Validation by qPCR. Eleven gene promoters identified by microarray as being differentially methylated were subjected to real-time PCR quantification of the enrichment by the MeDIP procedure. Results were normalized against a methylated luciferase gene-containing plasmid (control), which was added to every sample in equal quantity before MeDIP. The y-axis represents relative concentration levels generated by applying PCR to methylation-enriched DNA. Each real-time PCR reaction was performed in duplicate for all subjects. Shown are the averages per group. Error bars indicate the standard error of the mean. Above the chart are tracks of the regions with the microarray data. (The bars indicate the difference between the abuse and the non-abuse groups, bars descending from the physical map are regions that are more methylated in the abused than the non-abused group; lower tracks identify probes with the most statistically significant differences). Primers for each PCR are given in Additional file 3: Table S1. They were selected so that the forward primer (denote by ‘F’) binds to the left and the reverse primer (denoted by ‘R’) binds to the right of the most significantly different probe. In some cases, two sets of PCR primers were designed, denoted by ‘set1’ and ‘set2’. 85% of the eleven gene promoters show statistically significant PCR quantification differences (*: P<0.05; **: P<0.01), hence validating differences found by microarray. [file 1755-8794-7-13-S2.zip › 2875950191050941_fig7.tiff]

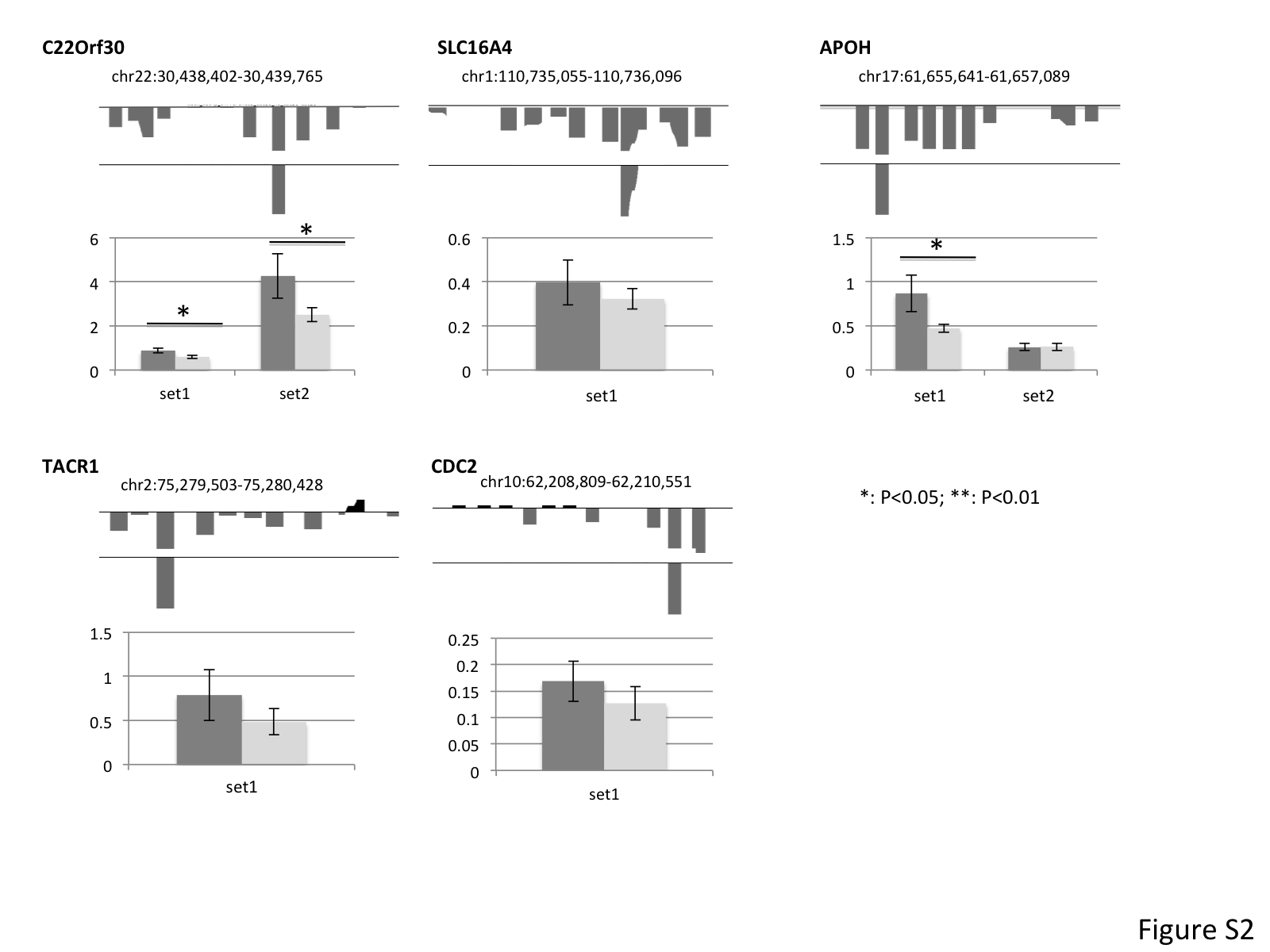

Supplement: Additional file 2: Figure S2 — Validation by qPCR. Eleven gene promoters identified by microarray as being differentially methylated were subjected to real-time PCR quantification of the enrichment by the MeDIP procedure. Results were normalized against a methylated luciferase gene-containing plasmid (control), which was added to every sample in equal quantity before MeDIP. The y-axis represents relative concentration levels generated by applying PCR to methylation-enriched DNA. Each real-time PCR reaction was performed in duplicate for all subjects. Shown are the averages per group. Error bars indicate the standard error of the mean. Above the chart are tracks of the regions with the microarray data. (The bars indicate the difference between the abuse and the non-abuse groups, bars descending from the physical map are regions that are more methylated in the abused than the non-abused group; lower tracks identify probes with the most statistically significant differences). Primers for each PCR are given in Additional file 3: Table S1. They were selected so that the forward primer (denote by ‘F’) binds to the left and the reverse primer (denoted by ‘R’) binds to the right of the most significantly different probe. In some cases, two sets of PCR primers were designed, denoted by ‘set1’ and ‘set2’. 85% of the eleven gene promoters show statistically significant PCR quantification differences (*: P<0.05; **: P<0.01), hence validating differences found by microarray. [file 1755-8794-7-13-S2.zip › 2875950191050941_fig8.tiff]

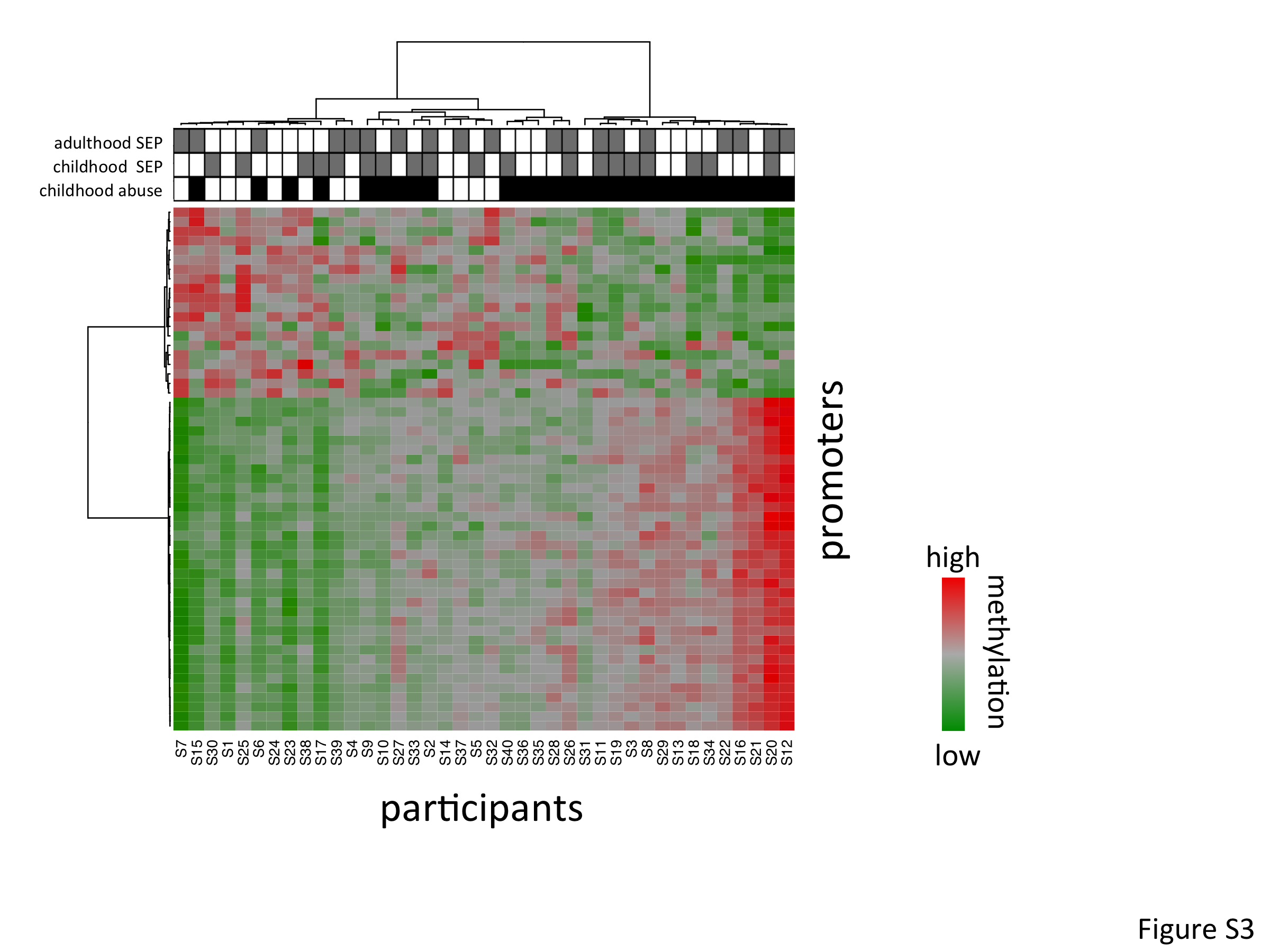

Supplement: Additional file 4: Figure S3 — Promoter methylation associated with childhood abuse. Heatmap showing MeDIP probe values from the 34 differentially methylated promoters (rows) across all 40 participants (columns) based on more stringent thresholds (q < 0.05 and p < 0.01, see Methods). Each promoter is represented by the probe most associated with childhood abuse. Blackened squares above the columns denote non-abuse males, white squares denote those with childhood abuse. Other covariates included are childhood and adulthood socio-economic position (white = low, gray = high). None appears to explain the main sample clusters. [file 1755-8794-7-13-S4.tiff]

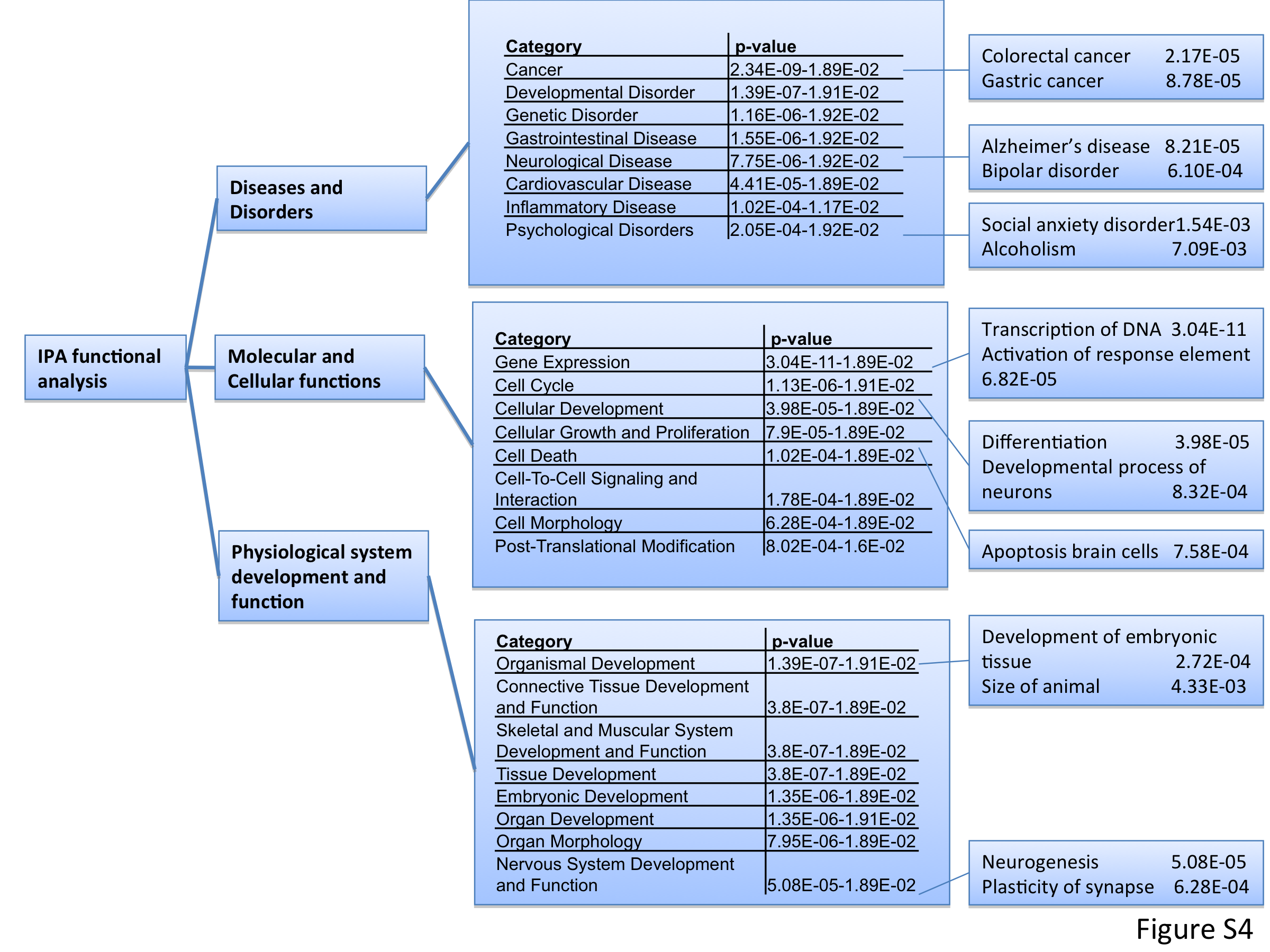

Supplement: Additional file 5: Figure S4 — Summary of functional analysis. Genes with hypermethylated or hypomethylated promoters in the abuse group were analysed by Ingenuity Pathway Analysis®. Gene categories enriched with this set of genes as well as enrichment p-values are listed. [file 1755-8794-7-13-S5.tiff]

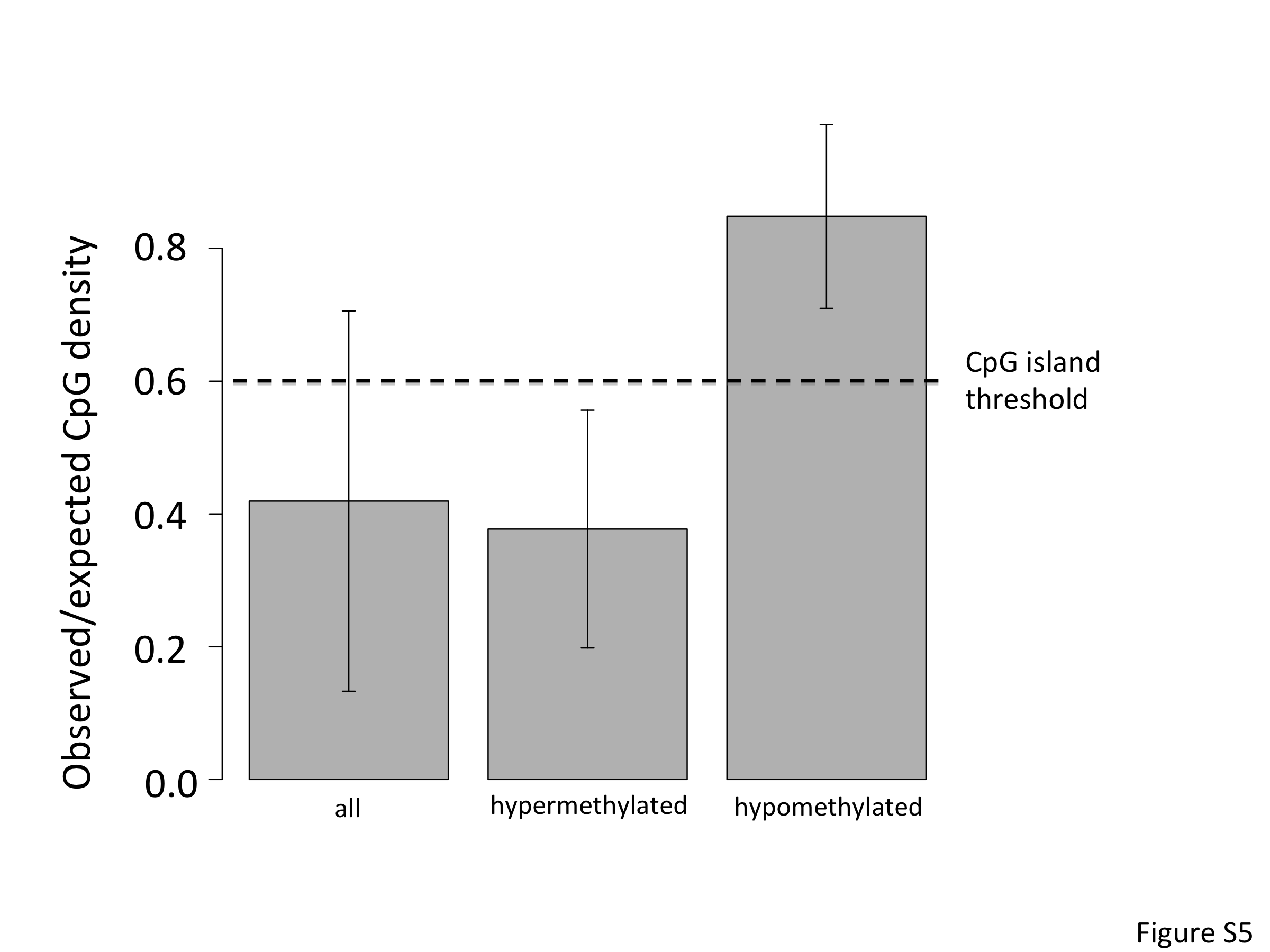

Supplement: Additional file 6: Figure S5 — CpG frequency in differentially methylated regions. Bars indicate average normalized CpG frequencies (observed/expected CpG frequency) of ‘all’ genomic regions profiled, regions ‘hypermethylated’ in abused individuals and regions ‘hypomethylated’ in abused individuals. Error bars depict standard deviation. The dashed line indicates the usual CpG frequency used to identify CpG islands. [file 1755-8794-7-13-S6.tiff]

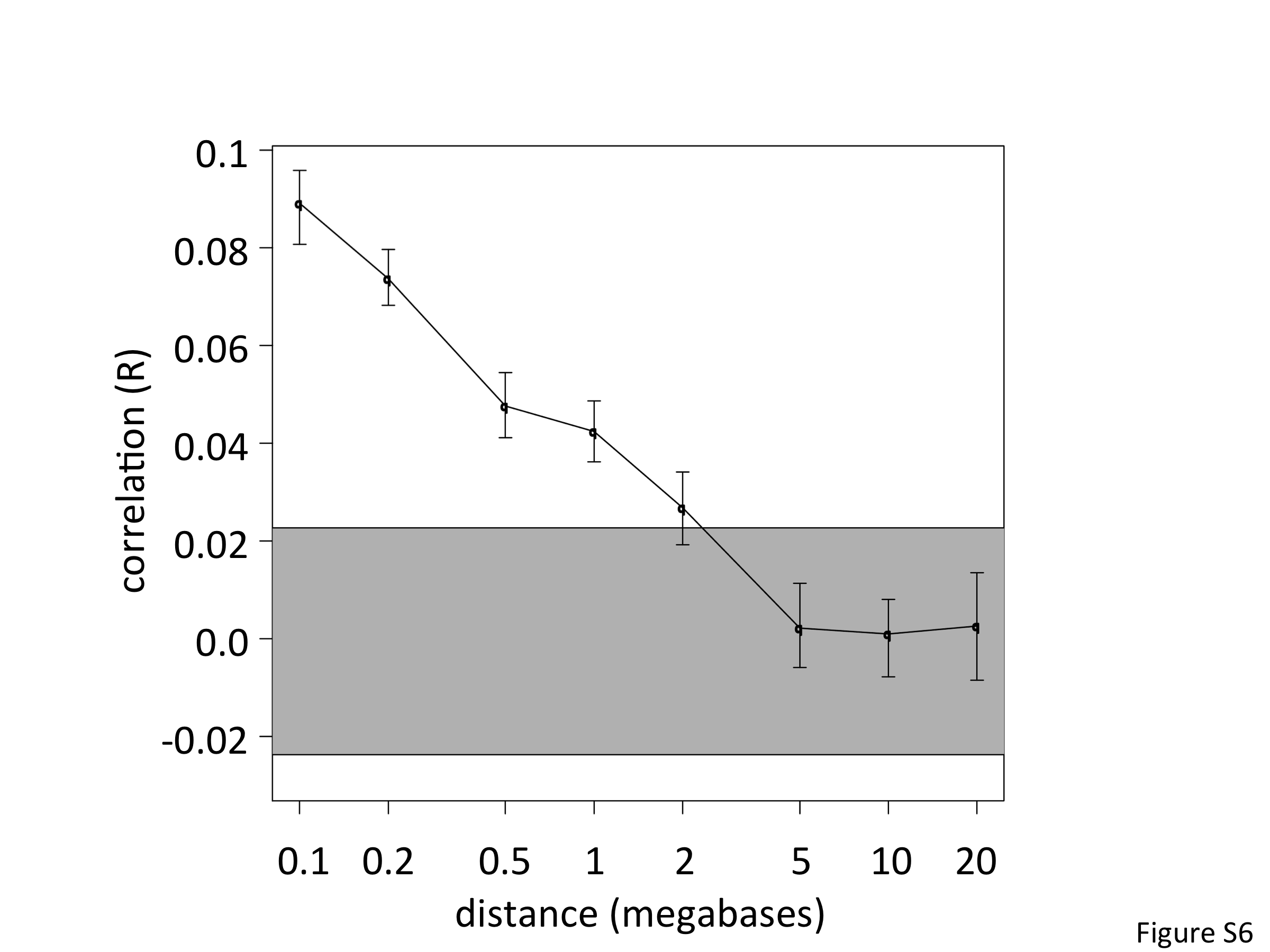

Supplement: Additional file 7: Figure S6 — Methylation dependencies across megabases. Shown are correlations of methylation differences from 500 kilobase regions at various distances apart. The level of clustering was quantified as the level of correlation between the differential methylation statistics within promoters at different distances apart. The solid grey region contains the 95% CI, and error bars contain the 95% CI for correlation values. [file 1755-8794-7-13-S7.tiff]
