# Supplementary material for: Childhood abuse is associated with methylation of multiple loci in adult DNA
Source: BMC Med Genomics. 2014 Mar 11;7:13. doi: 10.1186/1755-8794-7-13 (PMC4007631; doi:10.1186/1755-8794-7-13)
Supplement: Additional file 3 — Supplementary Material. [file 1755-8794-7-13-S3.doc]

**Supplementary Material**

***Study population*.** Participants were originally enrolled in the Perinatal Mortality Survey (PMS) of all born in England, Scotland and Wales, during one week in March 1958 with follow-up in childhood at 7, 11, 16y and in adulthood at 23, 33, 42y and 45y. 17,415 individuals were enrolled into the PMS from those eligible (N=17,638); immigrants with the same birth dates were recruited up to 16y (n=920), thus the total study population=18,558. At 45y, 11,971 participants still in contact with the study, and those who at 42y had not required a proxy interview, were invited to a clinical examination undertaken in their home by a trained nurse; 4,665 males and 4,712 females were seen September 2002-March 2004. From 4177 males providing consent to blood collection and DNA analysis, an eligible sample for epigenetic analysis was identified excluding: those reporting at 42 or 33y that they had had cancer (n=43); elevated (8mg/L) (n=125) or missing data (n=436) for C-reactive protein at 45y; immigrants (n=134) and others (n=39) who lacked perinatal data; and non-white (n=37) or missing data (n=1) on ethnicity. With respect to any influence of DNA variants on methylation profile differences, this cohort shows little population stratification.

This eligible sample (N=3362) was classified according to socio-economic position (SEP) in early childhood and mid-adulthood as follows.

A childhood SEP score was derived (range 1 (least) to 12 (most) disadvantaged) from cross-classification of:

1. father’s occupation in 1958. Six Registrar General’s (RG) occupational groups were used: professional (I), managerial/technical (II), other non-manual (IIInm), skilled manual (IIIm), partly skilled (IV) and unskilled manual (V), including those with no male head of household.
2. lacking or sharing access to household amenities (any of: hot water, bathroom and inside lavatory) or household overcrowding (>1 person /room) at age 7y vs others.

An adulthood SEP score was derived (range 1 (least) to 12 (most) disadvantaged) from cross-classification of:

1. the participant’s current or most recent occupation at 42y (or 33y if data were unavailable at 42y), categorized using the six RG groups mentioned above for father’s occupation.
2. housing tenure non-owner/buyer or financial difficulties at 45y (ascertained from two questions: how often do you not have enough money to afford clothing or food for you or your family, and how much difficulty do you have meeting the payment of bills. Men who sometimes/often or always had difficulties were identified) vs others.

For both childhood and adult SEP, scores were assigned whereby individuals with professional occupations and without housing/financial adversity had a score of 1 (i.e. least disadvantaged); professional occupation with housing/financial adversity was scored 2 and so forth to a score of 12 (most disadvantaged) for unskilled manual background and housing/financial adversity. Ranking individuals from the top and bottom quintiles of the distribution of SEP in both childhood and adulthood, we selected groups from those with sufficient blood DNA for methylation analysis. We selected 4 SEP groups: low SEP in both childhood and adulthood (LL n=10); childhood but not adulthood (LH n=11); adulthood but not childhood (HL n=11), and neither childhood nor adulthood (HH n=8). We then selected 12 who reported abuse, as described in the main text (7 low and 5 high child SEP; 7 low and 5 high adult SEP). Using the same SEP and abuse classifications we selected an additional 40 males for gene-specific replication.

**Genome-wide MeDIP analysis**

Details of the MeDIP analysis are presented elsewhere, and the steps taken in the microarray statistical analysis are summarized in Figure S1.

**Methodological considerations**

***Microarray analysis***

Statistical thresholds used in the microarray analysis are relaxed compared to those used in microarray analyses performed for other, especially cancer-related, studies. However, tumour generation involves major aberrations of cellular functioning and associated methylation alterations. Whereas, our study compares methylation profiles of DNA from blood cells that are functioning within normal variation and can be expected to have relatively subtle adjustments of their methylome. It is unlikely that this preliminary study of 40 selected participants can identify a definitive set of these adjustments. Our approach to obtaining promoter-level differential statistics breaks the assumption of the Wilcoxon rank-sum test that t-statistics from each promoter are independent. Dependence between t-statistics of neighbouring probes may arise from the fact that methylation levels of neighbouring CpG sites are often correlated and because DNA fragments binding to probes are typically longer than the average inter-probe spacing. Ideally, the promoter-level statistics would reflect the dependence between neighbouring CpG sites but exclude dependence due to DNA fragment size. In practice, this is not straight-forward and any proposed solution would require experimentation with samples of known but variable methylation levels. Such a study has yet to be performed and is beyond the scope of this paper. Nonetheless, our main hypothesis that there are methylation differences associated with childhood abuse in adult blood DNA can be answered without having to identify a definitive set of methylation differences associated with childhood abuse. Instead, it is only necessary to show that such a set exists. In view of these methodological considerations, our approach is to use relaxed statistical thresholds that could allow a limited number of false positives and then ask if the resulting set of differences show evidence of non-random organization, and finally, to validate selected differences using an alternative methylation profiling method (i.e. pyrosequencing) both in the original 40 DNA samples as well as in an independent set of samples, here for abuse and previously in relation to SEP.

***The potential influence of copy-number variation***

Due to the approach used, copy-number variation is less likely to explain normalized probe intensity variation than DNA methylation variation. This is because normalized probe intensities are derived by taking log2(Cy5 intensity/Cy3 intensity) where Cy5 intensity denotes the intensity due to methylated DNA fragments binding to the probe and Cy3 intensity denotes the intensity due to “input” or non-enriched DNA binding to the probe. In other words, the Cy3 intensity corresponds to DNA copy number. As a result, if there is a copy number increase, then in most cases both the Cy5 and Cy3 intensities will both increase by the same factor and be canceled out in the normalized probe intensity. The region on chr1:246250000-247000000 enriched with hypermethylated promoters perhaps deserves closer examination because it corresponds to a cluster of olfactory receptors which are known to be highly copy-number variable. We therefore tested the region for significant associations for Cy3 intensities with childhood abuse. However, none of these tests identified a probe with a p-value less than 0.05 and log2-fold difference between abuse groups greater than 0.25. Hence, the data do not support a copy-number association in this region with childhood abuse.

**Validation of MeDIP results and replication by bisulfite pyrosequencing.**

**PCR reagent concentrations.** DNA from participant samples was bisulfite-converted using conditions previously described. PCR amplifications of bisulfite-treated DNA consisted of two rounds because a single round did not yield sufficient amounts of specific product for pyrosequencing. Round1 template consisted of 40 ng of bisulfite-converted DNA and round2 template consisted of 2.0% (vol) round1 PCR products in 50 ul total reaction volumes. Final reagent concentrations were as follows: 1x Taq polymerase buffer (Fermentas/ThermoFisher Scientific), 1.5 mM MgCl_2_, 0.2 mM each dNTP, 0.5 uM each primer (F1 and R1, sequences below) and 2.5 U Taq polymerase. The same final concentrations were used for round2 except 0.1 uM each primer (F1 and R2, sequences below) was used. PCR primers were designed to measure CpG methylation on the positive strand. Both *SLC17A3* and *PM20D1* are coded on the negative strand. See Table S1 for primer sequences.

**PCR cycling.** For *SCL17A3*, Round1 PCR cycling conditions were: 95.0°C/5 min×1 cycle; (95°C/1.0 min, 50°C/2.5 min, 72°C/2.0 min)×40 cycles; 72°C/5.0 min×1 cycle. Round2 PCR cycling conditions were: 95°C/5.0 min×1 cycle; (95°C/1.0 min, 50°C/2.5 min, 72°C/1.0 min)×40 cycles; 72°C/5 min×1 cycle. For *PM20D1*, Round1 PCR cycling conditions were: 95.0°C/5 min×1 cycle; (95°C/1.0 min, 63.6°C/2.5 min, 72°C/2.0 min)×40 cycles; 72°C/5.0 min×1 cycle. Round2 PCR cycling conditions were: 95°C/5.0 min×1 cycle; (95°C/1.0 min, 58.4°C/2.5 min, 72°C/1.0 min)×40 cycles; 72°C/5 min×1 cycle.

**Optimization of PCR amplification conditions.** Twenty five round1/round2 combinations of annealing temperatures on HEK 293-derived bisulfite-treated DNA were tested. Five of these resulted in strong signal-to-noise ratios, and they were then tested on fully methylated *in vitro* (100%) and unmethylated (0%) HEK 293 DNA obtained by applying whole-genome amplification (WGA) to cell line DNA. Resulting PCR product samples were electrophoresed in 2% agarose gels and measured for abundance using spot densitometry (AlphaImager). Three combinations produced equal or near-equal PCR product abundance for the 0 and 100% methylated HEK WGA DNA, these were then tested for methylation frequency via pyrosequencing. For *SLC17A3*, one combination, 50˚C/50˚C, achieved methylation frequencies closest to those expected for the 0, 50 and 100% methylated controls; for *PM20D1*, one combination, 63.6˚C/58.4˚C, achieved methylation frequencies closest to those expected for the methylated controls. These combinations were then used for testing *SLC17A3* and *PM20D1*, respectively, in participant samples.

**Pyrosequencing.** 20 ul PCR product was used per pyrosequencing. See Table S2 for sequencing primers (SP).

**Table S1. Primers used in validation by quantitative PCR of differentially methylated regions in 11 genes**.

| Gene | Set | F or R | Primer Sequence (5'-3') |
| --- | --- | --- | --- |
| PM20D1 | 1 | F | TAGGTGGGTGAAGGGGACCCGG |
|  |  | R | GCATGGCTCAGCGGTGCGTTT |
|  | 2 | F | GCTGTCAGGCTACCGGGGTAGT |
|  |  | R | GGATCCTGGGCGGGGTCATTC |
| RUFY1 | 1 | F | TTGGACAGCTGCAAACCAAAAACAA |
|  |  | R | AGATCCCAAAGTAAGAGTCCTGGCA |
|  | 2 | F | CGGCTGGCACTATCCGGCAA |
|  |  | R | GCGTCCCGCCCCCAAACATT |
| NACA2 | 1 | F | TGGAATTCAGCCTCGGTTTTCTCAC |
|  |  | R | TGCGTTCAGATTGCTTCATGAGCA |
| MIND1 | 1 | F | CCACAGCGCCCAACCGTACTTC |
|  |  | R | TCATAGCAAAAGCTCAGGCCCCTG |
|  | 2 | F | GGTAGCGGCTCGGGAGAAGATGA |
|  |  | R | TCCTGTCTGGGGCGGACTGGAT |
| PPP2R5C | 1 | F | TGTTGGTGTGAAGAAAGGAGG |
|  |  | R | GAGTATTGCAGGATCCCATGAG |
|  | 2 | F | ATGTCAGCATATTTGCAATACAAAA |
|  |  | R | CTTACGTAGAAAATGAAAGCCGC |
| SLC17A3 | 1 | F | CAGGATCTCGGCGGTCAGGTGCTC |
|  |  | R | GTCCCAGACTTGGTCTTCCCTGGCC |
|  | 2 | F | GTCTCCTGCGAAGTAGCCCCC |
|  |  | R | GTTCTGCTTTGCTTGGATGAGGCAC |
| C22Orf30 | 1 | F | GTGCGCTTTTGTGGGGATGGGAT |
|  |  | R | CCCCTCCTCTGCTGGGTGTGA |
|  | 2 | F | TGTCAGCCTTCGGTTGGTTCTGA |
|  |  | R | GGCGATGGCGATCCCGTGAG |
| SLC16A4 | 1 | 2 | AGGGCTCAGACGATTTAGGCCCTC |
|  |  | 2 | TGCTTTTGAGCCTGCGGCAAAAA |
| APOH | 1 | F | CCGTCCTGCAATAGCAACATGGCA |
|  |  | R | AGCGCTTTCATATTTGGCTCTGTCT |
|  | 2 | F | TTGTTTCCGGGGTTGCCTGGAC |
|  |  | R | CAAGGCTGTGAGTGAAACAGGAGCT |
| TACR1 | 1 | 2 | TGGAGACAGCATCTCTCTTGCGGT |
|  |  | 2 | CACGCAGCACAGGGCTTTGC |
| CDC2 | 1 | 1 | CACGTTTCCAATGTCTCAGGAGTCA |
|  |  | 1 | TGTAAAGCATTTTCCACAGTGCCT |

**Table S2. Sequencing primers used in validation of differentially methylated regions by pyrosequencing**.

| Gene | Pos | Sequence (5'-3') | Remarks |
| --- | --- | --- | --- |
| SLC17A3 | F1 | GGGTTGTTAGTTAGTTTTAGGATTT |  |
|  | R1 | ATAACCTCTACTTTTAATTTAACATTAAAA |  |
|  | R2 | CTTTTAATTTAACATTAAAAAAAACTACTT | contains a 5'-biotin tag |
|  | SP1 | GGGTTGTTAGTTAGTTTTAGGATTT |  |
|  | SP2 | GCATAGATGCCTTTG |  |
| PM20D1 | F1 | TTATATATGAGTATAGGTGGGTGAAGG |  |
|  | R1 | AAAAACAAAACTAAAACCAAAAAAC |  |
|  | R2 | ACTATACTACTCCTAATTTTCCCTACC | contains a 5'-biotin tag |
|  | SP1 | TTATATATGAGTATAGGTGGGTGAAGG |  |
|  | SP2 | GTTGAATTGAGAAGGGAT |  |

**References**

1. Genome-wide association study of 14,000 cases of seven common diseases and 3,000 shared controls. Nature. 2007;447(7145):661-78. Epub 2007/06/08.

2. Borghol N, Suderman M, McArdle W, Racine A, Hallett M, Pembrey M, et al. Associations with early-life socio-economic position in adult DNA methylation. International Journal of Epidemiology. 2011.
